# Compared to X-ray, three-dimensional computed tomography measurement is a reproducible radiographic method for normal proximal humerus

**DOI:** 10.1186/s13018-016-0417-7

**Published:** 2016-07-15

**Authors:** Xiaoyang Jia, Yanxi Chen, Minfei Qiang, Kun Zhang, Haobo Li, Yuchen Jiang, Yijie Zhang

**Affiliations:** Department of Orthopaedic Trauma, East Hospital, Tongji University School of Medicine, 150 Jimo Road, 200120 Shanghai, China

**Keywords:** Three-dimensional, Shoulder morphology, Shoulder geometry, Shoulder anatomy, Measurement, Computed tomography, Computer-assisted

## Abstract

**Background:**

Accurate comprehension of the normal humeral morphology is crucial for anatomical reconstruction in shoulder arthroplasty. However, traditional morphological measurements for humerus were mainly based on cadaver and radiography. The purpose of this study was to provide a series of precise and repeatable parameters of the normal proximal humerus for arthroplasty, based on the three-dimensional (3-D) measurements.

**Methods:**

Radiographic and 3-D computed tomography (CT) measurements of the proximal humerus were performed in a sample of 120 consecutive adults. Sex differences, two image modalities differences, and correlations of the parameters were evaluated. Intra- and inter-observer reproducibility was evaluated using intraclass correlation coefficients (ICCs).

**Results:**

In the male group, all parameters except the neck-shaft angle of humerus, based on 3-D CT images, were greater than those in the female group (*P* < 0.05). All variables were significantly different between two image modalities (*P* < 0.05). In 3-D CT measurement, all parameters expect neck-shaft angle had correlation with each other (*P* < 0.001), particularly between two diameters of the humeral head (*r* = 0.907). All parameters in the 3-D CT measurement had excellent reproducibility (ICC range, 0.878 to 0.936) that was higher than those in the radiographs (ICC range, 0.741 to 0.858).

**Conclusions:**

The present study suggested that 3-D CT was more reproducible than plain radiography in the assessment of morphology of the normal proximal humerus. Therefore, this reproducible modality could be utilized in the preoperative planning. Our data could serve as an effective guideline for humeral component selection and improve the design of shoulder prosthesis.

## Background

Shoulder arthroplasty has been widely used for the treatment of glenohumeral osteoarthritis and severity fractures of the proximal humerus, which achieved positive clinical outcomes [1, 2]. It has been reported that restoration of the normal proximal humeral anatomy with prosthesis was very important for the postoperative clinical outcomes [1, 3]. To our knowledge, anatomic reconstruction begins with accurate comprehension of the morphological characteristics of the normal humerus.

Previous studies indicated that the morphology of the proximal humerus was considerably variable [4–10]. Meanwhile, a small mismatch between the natural humerus and prosthesis may lead to great changes in biomechanics [8, 11–14]. The normality of the humeral anatomy has been evaluated with traditional methods, which were mainly based on cadaver specimens [5, 6] and radiographs [8–10]. These studies [5, 6, 8–10] provided important reference values for the design of shoulder prosthesis. Despite the results being accurate, it was hard to obtain massive cadaver specimens, which may influence the sample size. Radiographs, based on two-dimensional plane, may be affected by the position of the humerus and the different projective angle of the tube. However, no published literature has reported whether there was a difference between 3-D CT and radiograph for measurement of the normal proximal humerus. A few studies [4, 7] recently have analyzed the normal anatomy of the proximal humerus with 3-D CT, but clear consensus of the measurements in 3-D space has not been reached. Thus, further research in this area is necessary.

The goals of this study were (1) to measure the 3-D morphological characteristics of the normal proximal humerus with novel 3-D techniques; (2) to examine whether there was a difference in the measurements between plain radiography and 3-D CT images; and (3) to evaluate the reproducibility of the measurement in the two imaging modalities.

## Methods

### Participant population

From December 2010 to January 2015, 230 participants were retrospectively involved in this study. Sixty-eight participants were excluded because of the presence of humeral fractures, humeral deformities, shoulder osteoarthritis, or previous humeral trauma, which were diagnosed by a musculoskeletal radiologist and a fellowship-trained orthopedist. For lacking of shoulder radiographs or CT records, 42 participants were not selected. Therefore, the CT and radiographic data of the normal humerus from 120 consecutive adults were analyzed in the study. There were 54 males and 66 females with the mean age of 52.7 ± 14.1 years (range, 19 to 69 years). The research protocol was approved by the Committee of the Medical Ethics of the hospital, and written informed consent was obtained.

### Radiology technique and image post-processing

The image data, collected from the Department of Radiology, were extracted in the Digital Imaging and Communication in Medicine (DICOM) 3.0 format (.dcm). Axial CT scans were performed with a 16-detector spiral CT scanner (GE Light-Speed CT; Waukesha, WI, USA). The thin-section CT images of all participants were input into the computer-aided orthopedic clinical research platform (SuperImage orthopedics edition 1.1, Cybermed Ltd, Shanghai, China) [15, 16].

**Fig. 1:**
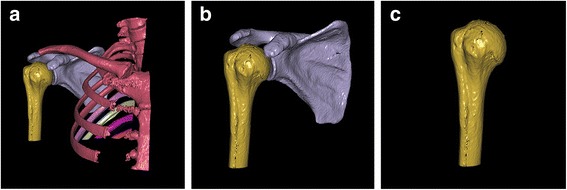
The process of generating the 3-D structure of the proximal humerus. **a** The humerus, scapula, clavicle, and other bones were extracted by 3-D interactive and automatic segmentation technique after SSD reconstruction, and different colors were assigned to the different bones. **b** Proximal humerus and scapula were marked *yellow* and *gray*, respectively, and other bones were deleted. **c** The proximal humerus was extracted solely

In this system, the 3-D images of each proximal humerus and its surrounding bones were generated by surface shaded display (SSD) algorithm with a reconstruction interval of 0.625 mm. All bone components were distinguished by performing 3-D interactive and automatic segmentation technique. Different colors were assigned to the different bones. Then, the proximal humerus was generated after removing the unrelated bones (Fig. 1).

The anatomic parameters of the proximal humerus were measured in the plain film radiography and in the 3-D CT images, respectively.

### Proximal humerus measurements

Before measurement, the following points, lines, and planes were defined and matched on the proximal humerus:

1. Humeral shaft axis (HSA): as previously reported [4], the humeral shaft (from metaphysis to the deltoid tuberosity) was approximated as a cylinder, which best fitted the shape of the upper humerus. Two points (point A and B) were the midpoint of the diameter of humeral shaft. The line passing through the two points was defined as the HSA (Fig. 2a).

**Fig. 2:**
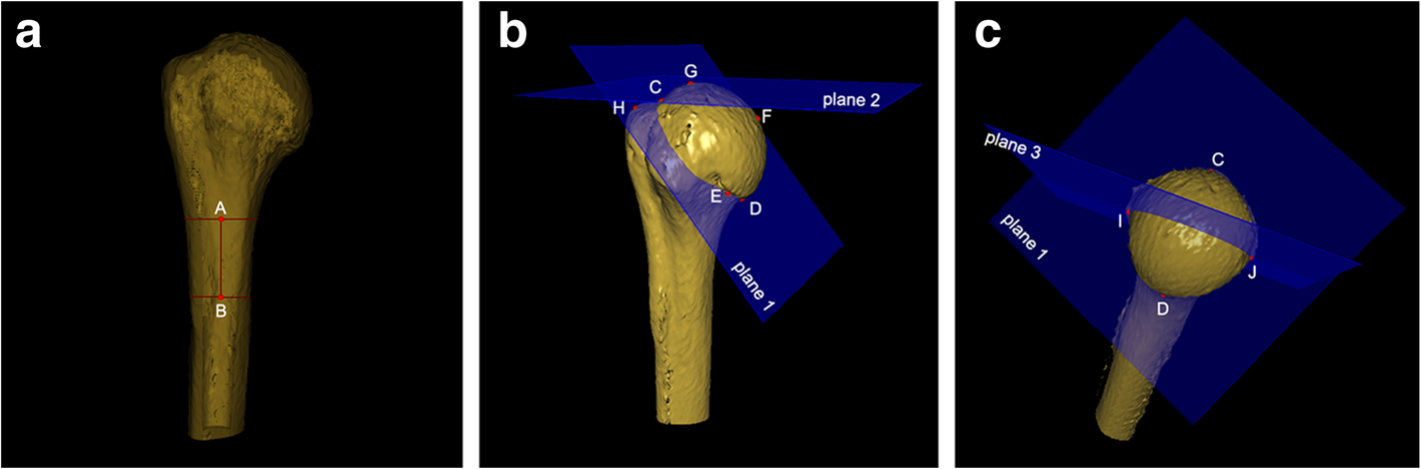
Definition of point, line, and plane of proximal humerus. **a** Line *AB* = humeral shaft axis (HSA) in perspective mode. **b** *C* = most superior point of articular surface; *D* = most inferior point of articular surface; *E* = concave point of articular surface; *F* = furthest vertical distance point on articular surface to plane 1; *G* = most superior point of humeral head; *H* = most superior point on greater tuberosity; *plane 1* = anatomical neck plane; *plane 2* = the plane that was via point *G* and parallel to the transverse axis of humeral shaft. **c** *I* and *J* = intersection of *plane 1* and *plane 3* on the articular surface; *plane 3* = axial plane that was the vertical plane through the midpoint of line CD
2. Point C was defined as the most superior point of the articular surface at the insertion of the supraspinatus tendon. And its corresponding lowest point of the articular surface was point D, as previously described [17] (Fig. 2b).
3. Point E was a point of the relatively concave portion on the articular surface. The anatomic neck plane (plane 1) was determined by point C, D, and E (Fig. 2b).
4. The point (point F) on the articular surface had the furthest vertical distance to plane 1 (Fig. 2b).
5. Points G and H were defined as the most superior point of the articular surface and the most superior point on the greater tuberosity, respectively. Plane 2 was defined as the plane that was via point G and parallel to the transverse axis of the humeral shaft (Fig. 2b).
6. Plane 3 was the vertical plane through the midpoint of line CD. Point I and J were defined as the intersection of plane 1 and plane 3 (axial plane) on the articular surface (Fig. 2c).

**Fig. 3:**
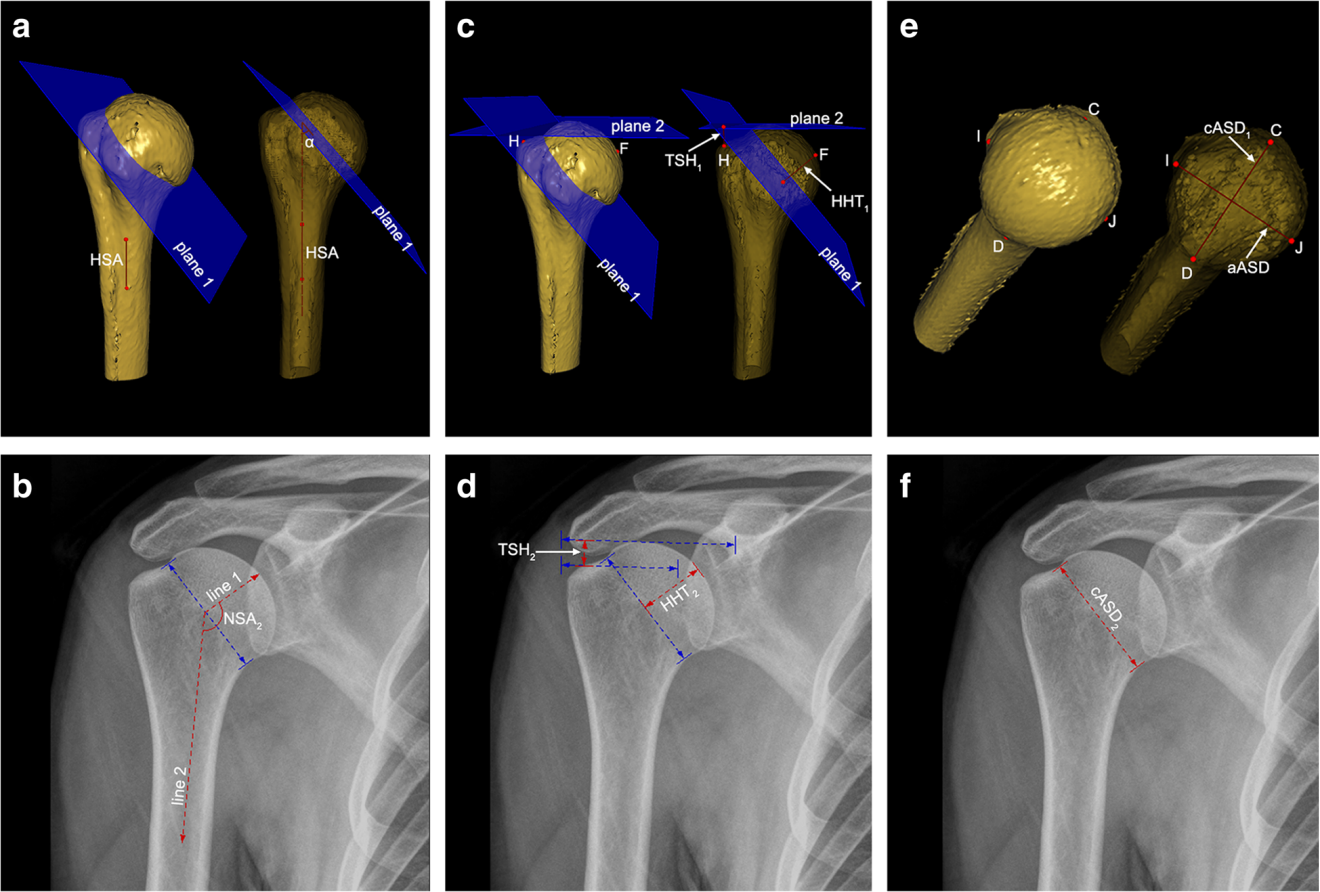
Morphological measurements of the proximal humerus. **a** *NSA*_*1*_ = *α* + 90°; *α* = angle between *plane 1* and HSA. **b** *NSA*_*2*_ = angle between *line 1* and *line 2*; *line 1* was perpendicular to anatomic neck; *line 2* was parallel to the long axis of the humeral shaft. **c** *HHT*_*1*_ = perpendicular distance from point *F* to *plane 1*; *TSH*_*1*_ = perpendicular distance from point *H* to *plane 2*. **d** *HHT*_*2*_ = the longest perpendicular distance between head surface and anatomic neck; *TSH*_*2*_ = perpendicular distance between two lines. **e** *cASD*_*1*_ = distance between C and D; *aASD* = distance between *I* and *J*. **f** *cASD*_*2*_ = length of anatomic neck. *NSA* neck shaft angle; *HAS* humeral shaft axis; *HHT* humeral head thickness; *TSH* tuberosity-to-articular surface height; *cASD* articular surface diameter in the coronal plane; *aASD* articular surface diameter in the axial plane

The morphological parameters of the proximal humerus were measured included the following (Fig. 3):

1. The neck-shaft angle (NSA): in the 3-D images (NSA_1_), it was calculated by 90° plus the head inclination angle (α), which was the angle between plane 1 and HSA (Fig. 3a). In the anteroposterior (AP) view radiographs (NSA_2_), it was measured by the intersection of the line parallel to the long axis of the humeral shaft and the line vertical to the anatomic neck (Fig. 3b), as previously described [6].
2. The humeral head thickness (HHT): it was, in the 3-D images (HHT_1_), the perpendicular distance from point F to plane 1 (Fig. 3c). In the AP view radiographs (HHT_2_), it was defined as the longest vertical distance between the articular surface and the anatomic neck (Fig. 3d).
3. The tuberosity-to-articular surface height (TSH): it was, in the 3-D images (TSH_1_), the perpendicular distance from point H to plane 2 (Fig. 3c). In the AP radiographs (TSH_2_), it was defined as the vertical distance between the tangent line of the highest point of the greater tuberosity and the articular surface (Fig. 3d).
4. The articular surface diameter (ASD): it was the diameter of the head segment at the anatomic neck plane. It was equal to the distance between C and D (in coronal plane, cASD_1_) and between I and J (in axial plane, aASD) in the 3-D images (Fig. 3e). In the AP view radiographs (cASD_2_), it was the length of the diameter of the anatomic neck (Fig. 3f).

### Statistical analysis

Statistical analysis was performed using SPSS (version 19.0, Chicago, IL, USA). On the basis of Bonett [18], intra-observer reproducibility and inter-observer reproducibility were evaluated in 36 participants randomly selected using intraclass correlation coefficients (ICCs). The measurement was performed independently by three surgeons. All 120 subjects were measured by the main examiner. With a 3-week interval, 36 selected subjects were measured again by the main examiner and one time separately by the other examiners. The average ICC of inter-observer reproducibility was determined by the measurements of the three examiners.

All parameters were examined for normality using of the Kolmogorov-Smirnov test and were found to follow the normal distribution. Thus, data were reported as mean ± standard deviation (SD). Sex differences were compared using independent samples *t* tests. NSA, TSH, HHT, and cASD were compared between the 3-D CT and the AP view radiographs by paired sample *t* tests. Pearson correlation coefficients were performed among NSA_1_, TSH_1_, HHT_1_, cASD_1_, and aASD. The level of significance was defined as *P* < 0.05 for all analyses.

## Results

The mean NSA_1_ was 132.1° ± 4.4° (range, 120.5° to 142.6°), and the mean NSA_2_ was 133.0° ± 5.2° (range, 122.1° to 143.2°). The mean TSH_1_, TSH_2_, HHT_1_, HHT_2_, cASD_1_, cASD_2_, and aASD were 7.2 ± 2.3 mm (range, 3.8 to 14.9 mm), 10.0 ± 2.7 mm (range, 5.0 to 17.1 mm), 18.8 ± 2.2 mm (range, 10.3 to 23.3 mm), 23.3 ± 2.3 mm (range, 18.0 to 28.0 mm), 44.2 ± 4.1 mm (range, 36.3 to 52.3 mm), 46.4 ± 4.9 mm (range, 38.0 to 57.1 mm), and 40.4 ± 3.6 mm (range, 32.1 to 47.4 mm), respectively.

In the male group, the values of HHT_1_, HHT_2_, cASD_1_, cASD_2_, and aASD were greater than those in the female group (*P* < 0.001). In 3-D images, males had a significantly larger TSH than females had (*P* = 0.047), but not in radiographs (*P* = 0.350). No significant gender difference was observed in NSA_1_ and NSA_2_ (*P* = 0.142; *P* = 0.092) (Table 1).

**Table 1:** Anatomical parameters of the proximal humerus

	Mean ± SD			Sex difference	
	Total	Male	Female	*t* value	*P* value
3-D CT scans					
NSA_1_ (°)	132.1 ± 4.4	131.4 ± 3.9	132.6 ± 4.8	−1.480	0.142
TSH_1_ (mm)	7.2 ± 2.3	7.7 ± 2.6	6.8 ± 2.1	2.010	0.047*
HHT_1_ (mm)	18.8 ± 2.2	20.1 ± 1.6	17.7 ± 2.0	7.040	<0.001*
cASD_1_ (mm)	44.2 ± 4.1	47.3 ± 2.3	41.7 ± 3.4	10.472	<0.001*
aASD (mm)	40.4 ± 3.6	43.1 ± 2.2	38.2 ± 2.9	10.298	<0.001*
Radiographs					
NSA_2_ (°)	133.0 ± 5.2	132.1 ± 5.6	133.7 ± 4.7	−1.696	0.092
TSH_2_ (mm)	10.0 ± 2.7	10.1 ± 3.0	9.7 ± 2.4	0.938	0.350
HHT_2_ (mm)	23.3 ± 2.3	24.4 ± 1.9	22.4 ± 2.1	5.415	<0.001*
cASD_2_ (mm)	46.4 ± 4.9	49.8 ± 4.0	43.6 ± 3.6	8.866	<0.001*

*SD* standard deviation, *NSA* neck-shaft angle, *TSH* tuberosity-to-articular surface height, *HHT* humeral head thickness, *cASD* articular surface diameter in the coronal plane, *aASD* articular surface diameter in the axial plane

*Statistically significant (*P* < 0.05)

All variables were significantly different between 3-D CT images and radiographs (*P* < 0.05) (Table 2).

**Table 2:** Comparison of parameters in different image modalities

	*t* value	*P* value
3-D CT/radiographic		
NSA_1_/NSA_2_	−2.080	0.040*
TSH_1_/TSH_2_	−14.075	<0.001*
HHT_1_/HHT_2_	−22.288	<0.001*
cASD_1_/cASD_2_	−6.877	<0.001*

*NSA* neck-shaft angle, *TSH* tuberosity-to-articular surface height, *HHT* humeral head thickness, *cASD* articular surface diameter in the coronal plane

*Statistically significant (*P* < 0.05)

The correlation among all parameters of the 3-D images was listed (Table 3). NSA_1_ was only correlated with TSH_1_ (*r* = 0.586). All parameters, except NSA_1_, had correlation with each other (*P* < 0.001), particularly between the two diameters of the humeral head (*r* = 0.907 and *P* < 0.001).

**Table 3:** Correlations among parameters in 3-D CT images

Parameters	TSH_1_	HHT_1_	cASD_1_	aASD
NSA_1_	*r* = 0.586	*r* = 0.106	*r* = 0.020	*r* = 0.036
	*P* < 0.001*	*P* = 0.248	*P* = 0.831	*P* = 0.695
TSH_1_		*r* = 0.391	*r* = 0.361	*r* = 0.413
		*P* < 0.001*	*P* < 0.001*	*P* < 0.001*
HHT_1_			*r* = 0.705	*r* = 0.681
			*P* < 0.001*	*P* < 0.001*
cASD_1_				*r* = 0.907
				*P* < 0.001*

*r* correlation coefficient, *NSA* neck-shaft angle, *TSH* tuberosity-to-articular surface height, *HHT* humeral head thickness, *cASD* articular surface diameter in the coronal plane, *aASD* articular surface diameter in the axial plane

*Statistically significant (*P* < 0.05)

**Fig. 4:**
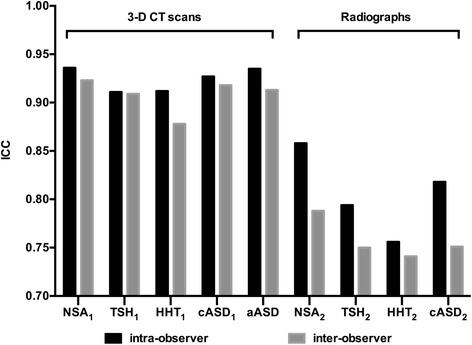
Intra-observer and inter-observer reproducibility of 3-D CT and radiographic anatomic measurements. *ICC* intraclass correlation coefficient; *NSA* neck-shaft angle; *TSH* tuberosity-to-articular surface height; *HHT* humeral head thickness; *cASD* articular surface diameter in the coronal plane; *aASD* articular surface diameter in the axial plane

Intra-observer and inter-observer reproducibility of all variables ranged from 0.741 to 0.936. All ICCs about the 3-D CT measurement exceeded 0.8, indicating excellent agreement. The agreement of the 3-D CT measurement (ICC range, 0.878 to 0.936) was higher than the agreement of the radiographs (ICC range, 0.741 to 0.858) (Fig. 4).

## Discussion

The reconstruction of the normal anatomy is the goal of shoulder arthroplasty. Therefore, it is important to have a comprehensive understanding about the normal morphological characteristics of the humerus. Compared to plain radiography, 3-D CT was more reproducible. Its superiorities in anatomic measurement had been proved by previous studies [19–21]. Holme et al. [21] demonstrated that 3-D CT was superior in the evaluation of the orientation of the tibial component (ICC range, 0.69 to 0.99). This study also obtained the similar “results”. The examiners obtained all excellent intra- and inter-observer reproducibility using 3-D CT in the assessment of anatomic parameters (ICC range, 0.878 to 0.936). These morphological parameters would be helpful to decide implant’s size, position, and design, when anatomic parameter measurement of the proximal humerus was based on the scientific method [16].

The value of the neck-shaft angle is completely relative, which is a research focus in the morphology of the proximal humerus. Jeong et al. [22] measured cadaveric humeri and revealed that the mean neck-shaft angel was 134.7°, ranged from 115° to 148°. Takase et al. [10], using plain radiographs, found that the mean NSA was 140.5°. Matsumura et al. [7], using CT images, relied on 160 shoulders and demonstrated that the mean angle was 135°. This study, based on 3-D CT, showed that the mean neck-shaft angle was 132.1°. This difference may be due to either the differences in race [7] or measuring technique. The present study implied that the neck-shaft angle was larger in plain radiographs than in CT images, which was consistent with the previous findings [10]. Approximately 20 % of the normal participants had an excessive valgus (>140°) or varus (<130°) neck-shaft angle [22]. For the fixed-angled prosthesis, if the natural humerus has an excessive valgus or varus neck-shaft angle, the osteotomy line should start from the inferomedial margin of the humeral head and from the superolateral margin, respectively [22, 23]. Previous study revealed that neck-shaft angle differences between the natural humerus and prosthesis cannot be corrected by adjusting humeral head thickness [9], which correlated well with the results of this study that there was no correlation between them. For the wide range of the NSA, Jeong et al. [22] noted that the adjustable-head prosthesis had better adaptability than the fixed-head device. It has been reported that an increase of the NSA may be associated with subacromial impingement or the alteration in the kinematics of the shoulder [8, 12]. Takase et al. [10] found that the uniform setting of the TSH may cause dysfunction of the abductor muscles without consideration of the neck-shaft angle in prosthetic reconstruction. The variability of the NSA may be the reason for the large overall range of the TSH [10], which agreed with our findings that NSA had correlation with TSH. Thus, its considerable variation in individuals is suggested to be kept in mind.

Restoring the normal tuberosity-to-articular surface height and head thickness by means of shoulder arthroplasty provided the well-functional results. Early clinical investigation [1] demonstrated that postoperative subacromial impingement was a common complication after arthroplasty, which may be caused by prominent great tuberosity relative to the humeral head prosthesis. Nyffeler et al. [14] indicated that if the prosthesis head was placed too high relative to the tuberosity, the inferior capsule would be tightened and limit abduction, which could impair the shoulder function. For these reasons, whether the prosthesis head was positioned too high or too low to the greater tuberosity, dysfunctions of glenohumeral joint may be caused. A cadaveric biomechanical study by Harryman et al. [11] found that a change in head thickness (≥5 mm) may decreased the range of shoulder motion and resulted in translation of the humeral head on the glenoid with tightening of the capsule and rotator cuff. Early study [13] suggested that decreasing the head thickness would diminish excursion of the glenohumeral joint. Therefore, the standard value of TSH and HHT was crucial for intraoperative setting prosthesis and reduced the occurrence of postoperative dysfunctions.

It was found that the cASD_1_ was not as long as the aASD in the present study. The cASD_1_ and aASD, the diameter of head segment at the anatomic neck plane, were the important parameters of the humerus, which were closely related to the choice of prosthesis. It has been reported that the shape of the head segment at the anatomic neck plane was not spherical [13, 17, 24]. Harrold et al. [17] revealed that cASD_1_ was longer than aASD, and that the two diameters were with a high degree of correlation, which were consistent with the findings of this study. Due to the discrepancy between the two diameters, the selection of the prosthesis matching the diameter in the coronal plane and axial plane with the same length would result mismatch. When the diameter of the prosthetic head was too long, the protrusion would increase the tension of the subscapularis or infraspinatus, or both, which may cause tendinopathy and tendon rupture [17]. However, using a smaller size of the prosthetic head would reduce glenohumeral range of motion and cause translation of the humeral head on the glenoid. Meanwhile, it would lead to instability of the glenohumeral joint and accelerate wear of the glenoid [8, 11]. Therefore, using suitable prosthesis to match the humeral head may reduce the risk of aforementioned complications [8, 11, 17].

There are several limitations in this study. First, considering the extra dose of radiation, participants were not asked to receive other scans. Thus, the correlation of bilateral humerus for these parameters was not evaluated. Second, data of the height of participants were not analyzed that may have correlation with the parameters of the humerus. Third, the parameters of the glenoid were not measured, which also were the important references for the prosthesis. Last, this study was not being performed with cadavers, and the bones were not actually directly measured with calipers or goniometers. Therefore, the gold standard could not be obtained for comparison. Despite the limitations, we hope that the present study could give some clinical reference, when the design of shoulder prosthesis and the guideline for the humeral component selection are needed. Above all, further studies with comparison to a gold standard and better design would be required and helpful to confirm the accuracy of these measurements.

## Conclusions

This study demonstrated that 3-D CT was reproducible to assess the morphology of the normal proximal humerus, which could be used in the preoperative planning. The gender difference of parameters was found, and the strong correlation among them should be considered. We believe that our data can serve as an effective guideline for humeral component selection and improve the design of shoulder prosthesis.

## Abbreviations

3-D, three-dimensional; aASD, articular surface diameter in the axial plane; AP, anteroposterior; ASD, articular surface diameter; cASD, articular surface diameter in the coronal plane; CT, computed tomography; DICOM, digital imaging and communication in medicine; HAS, humeral shaft axis; HHT, humeral head thickness; ICCs, intraclass correlation coefficients; NSA, neck-shaft angle; SD, standard deviation; SSD, surface shaded display; TSH, tuberosity-to-articular surface height
